# Periostin promotes the proliferation and metastasis of osteosarcoma by increasing cell survival and activates the PI3K/Akt pathway

**DOI:** 10.1186/s12935-021-02441-6

**Published:** 2022-01-20

**Authors:** Chaojian Xu, Ziyue Wang, Long Zhang, Yi Feng, Jia Lv, Zhuangzhuang Wu, Rong Yang, Taiyong Wu, Jian Li, Ruhao Zhou, Zhi Tian, Junjun Bai, Huadong Zhang, Yanping Lan, Zhi Lv

**Affiliations:** 1 Shanxi Medical University, Taiyuan, China; 2 Department of Orthopaedics, The Second Hospital of Shanxi Medical University, No. 382 Wuyi Road, Taiyuan, 030000 Shanxi China; 3 Beijing Shijitan Hospital, Capital Medical University, Beijing, China; 4 Xiamen University, Xiamen, China

**Keywords:** POSTN (Periostin), Osteosarcoma, Proliferation, Metastasis, Pathway

## Abstract

**Background:**

Silencing of the periostin gene (*POSTN*) can inhibit the biological process of several different cancers, and this inhibition may be related to down-regulation of PI3K/AKT signaling. However, the effect of *POSTN* on the progression, proliferation, and invasion of osteosarcoma (OS) remain unclear.

**Methods:**

We used the Gene Expression Omnibus (GEO) database to screen datasets on in situ OS and lung metastases to identify core genes and potential pathways. We used additional bioinformatics tools to identify protein–protein interactions (PPIs) and gene networks, and selected the top seven genes whose expression had the strongest correlations with other genes.

**Results:**

The results indicated that *POSTN* was a major hub gene. Subsequent analysis of gene expression profiles showed that *POSTN* was highly expressed in 262 cases with sarcoma and expression was closely related to poor prognosis. We also performed enrichment analysis to identify differentially expressed genes and used real-time PCR, western blotting, and immunohistochemistry analyses to measure POSTN expression in cells and tissues. Transfection of a *POSTN*-shRNA plasmid into cultured OS cells (Saos-2) effectively inhibited the proliferation, invasion, and migration of these cells. Taken together, our results suggest that *POSTN* may play a role in promoting the proliferation and metastasis of OS by activation of the PI3K/Akt signaling pathway.

**Conclusions:**

Our results provide a preliminary characterization of the mechanism by which *POSTN* may regulate the migration and invasion of OS cells and also provide a theoretical basis for identifying biomarkers that have potential use for the diagnosis and treatment of OS.

## Background

Osteosarcoma (OS) is the most common primary malignant bone tumor. It has high rates of malignancy, invasion, recurrence, and metastasis, and occurs mostly in teenagers [1]. Treatments for OS have improved in recent years due to advances in adjuvant chemotherapy and surgery, but recurrence and metastasis remain major problems in the treatment of these patients. The local recurrence rate of OS after an operation is about 10 to 20%, and the survival rate after local recurrence is significantly reduced [2]. Metastasis, mainly to the lungs, occurs in almost half of patients with OS, and about 80% of patients with metastases die within 5 years [3]. However, control of tumor recurrence and metastasis are among the most difficult problems in treatment of these patients. Thus, further research is needed to focus on the pathogenesis of OS and to study the effects of neoadjuvant chemotherapy.

Periostin (POSTN) is a bone adhesion molecule first discovered by Takcshita et al. in mouse osteoblast cell line. It is a unique extracellular matrix protein that can promote the aggregation and differentiation of osteoblasts and their precursors in the periosteum [4]. POSTN occurs in connective tissues that have abundant collagen and plays a role in embryonic development and other normal biological activities, but it also functions in tissue injury and inflammation. POSTN has high expression in the periosteum and periodontal ligament, and is therefore also referred to as periosteal protein [5, 6]. POSTN also has high expression in a variety of malignant tumor-related interstitial cells, including non-small cell lung cancer, breast cancer, ovarian cancer, and thymoma [7]. POSTN functions in the migration and adhesion of normal cells, but also affects tumor cell proliferation, apoptosis, and invasion, and angiogenesis [8]. Previous studies reported that POSTN had high expression in OS tissues, and its level in blood was negatively correlated with patient prognosis [9]. Molecular studies reported that POSTN regulated vascular endothelial cells and angiogenesis via the PI3K/AKT signaling pathway [10–12].

High-throughput sequencing is now widely used and is an important tool in life sciences research. In this study, we selected two microarray datasets, GSE85537 (3 OS lung metastasis samples and 3 OS non-metastasis samples) and GSE37552 (2 OS lung metastasis samples and 2 OS non-metastasis samples), from the Gene Expression Omnibus (GEO) database to analyze differentially expressed genes in OS using multiple bioinformatics tools. We also examined the expression of POSTN in cultured OS cells and tissues at the gene and protein levels, and performed cell function and molecular biology experiments to examine the possible effect of POSTN on the proliferation and metastasis of tumor cells and the possible role of the PI3K/Akt pathway in these pathogenic processes. Our general purpose was to evaluate the feasibility of using POSTN as an indicator of malignant OS and to provide a theoretical basis for using tumor metastasis-related genes for guiding future clinical treatments of OS.

## Materials and methods

### Patients and specimens

OS and peritumor tissues from 8 patients were collected at the Second Hospital of Shanxi Medical University from January 2018 to December 2019 and stored in liquid nitrogen immediately after collection. The specimens were numbered according to the specimen bank, and names and personal information were strictly protected. All patients signed informed consent agreements from the Biological Specimen Bank of the Second Hospital of Shanxi Medical University. This study was approved by the Ethics Committee of the Second Hospital of Shanxi Medical University.

### Cell cultures and treatments

The three OS cell lines were Saos-2 (cultured in RPMI 1640 complete medium containing 10% FBS and 1% Mycillin), MG-63 (cultured in MEM complete medium containing 10% FBS and 1% Mycillin), and U2-OS (cultured in DMEM complete medium containing 10% FBS and 1% Mycillin). All cells were cultured in an incubator at 37 °C, 5% CO_2_, and 95% humidity.

### Cell transfection

About 4 × 10^4^ cells (1 mL) were incubated in six-well plates. Three accessory holes were established in each well, and transient transfection was performed on the next day. The experimental group was transfected with the pPLK-*POSTN*-shRNA, and the control group with pPLK-*Scramble*-shRNA. During transfection, plasmids were mixed with Lipofectamine 3000 and then placed into the holes. When the final concentration of shRNA in each group reached 100 nmol/L, cells were transferred to the incubator. After 24 h, normal culture medium was used for continuous culture. Transfection efficiency was assessed using fluorescence microscopy and qRT-PCR. The *POSTN-* shRNA plasmid (GenBank ID: 10631) sequence was GCAACGTGAATGTTGAATT, which is the best knockdown effect plasmid in the 3 target gene sequences for the shRNA plasmid of POSTN gene, and the *Scramble*-shRNA plasmid sequence was GTTCTCCGAACGTGTCACGTT.

### RNA extraction and real-time PCR

Trizol (Invitrogen, USA) was used to lyse cells for extraction of total RNA and the Bulge-LoopTM miRNA qRT-PCR Starter Kit was used for reverse transcription of RNA into cDNA. The SYBR Green qPCR Kit (Takara, Japan) was used for quantitative fluorescence measurements. According to the instructions, specimens were added to a 96-well plate, which was then placed in the IQ5 Multicolor Real-Time PCR Detection System (Bio-Rad Laboratories, Hercules, CA, USA) to initiate the reaction. For *POSTN*, the upstream primer sequence was: 5′-CCCCGTGACTGTCTATAAGC-3′ and the downstream primer sequence was: 5′-AAATGACCATCACCACCTTCA-3′. For *RS18* (internal reference), the upstream primer sequence was: 5′-GCCATCAAGGGTATCGGTAGAC-3′, and the downstream primer sequence was: 5′-CTGCCTGTTAAGGAACCAGTCAG-3′. The level of *POSTN* was calculated using the 2^−ΔΔCt^ method.

### Western blot analysis

The Tissue or Cell Total Protein Extraction Kit (KeyGEN, Nanjing, China) was used to extract total proteins from cells and tissues, and the BCA Protein Quantitative Kit (Boster, China) was used to measure total protein levels. Samples were then stored at low temperature prior to use. Each sample was mixed with the loading buffer and heated to 100 °C for 10 min. Then, 25 μg of protein was added to each lane of the gel, followed by electrophoresis and membrane transfer. The membrane was sealed and incubated overnight at 4 °C with different primary antibodies (POSTN rabbit anti-human, Proteintech, China, 1:1000; PCNA rabbit anti-human, ABclonal, China, 1:1000; P-AKT rabbit anti-human, ABclonal, China, 1:1000; AKT rabbit anti-human, ABclonal, China, 1:1000; or PI3K rabbit anti-human, ABclonal, China, 1:1000). TBST was used to wash the membrane 3 times (5 min each) and goat anti-rabbit IgG (Boster, China, 1:8000) with horse-radish peroxidase was then added and incubated at room temperature for 1 h. TBST was used to wash the membrane 3 times (5 min each), and the ECL reagent was then added for visualization.

### Cell invasion and wound healing assays

For the cell invasion assay, at 48 h after cell transfection, trypsin was added for digestion and a polycarbonate microporous membrane was then placed between the upper and lower chambers (hole diameter: 8 μm). About 500 μL of complete medium containing 10% serum was added into the lower chamber and 5 × 10^4^ cells (100 µL) were added to the upper chamber of Matrigel and cultured in an incubator at 37 °C for 12 h. Then, cells in the upper chamber were collected and fixed with 4% paraformaldehyde for 30 min, dyed with 0.1% crystal violet for 20 min, followed by addition of hematoxylin. The transmembrane cells were counted under a microscope by examination of 5 random fields to obtain an average.

For the wound healing assay, cells were added to 6-well plates (density: 1.5 × 10^5^ cells/mL) and then placed in an incubator for 24 h. A 100 µL pipette tip was placed perpendicular to the surface to make scratches. The cells were then washed with a PBS solution, the wounded and necrotic cells were removed, and the remaining cells were added to medium containing 10% serum and placed in an incubator. Photos were taken at 24 h and 48 h to record closure of the wounded area.

### lmmunohistochemistry

Paraffin specimens of OS and adjacent normal bone tissues were sectioned, dewaxed, and dehydrated. The sodium citrate thermal method was used for slicing, and the enzyme antigen repair method was used for the normal bone tissues. According to the instructions of the Immunostain SP Kit (ZSGB-BIO, Beijing, China), the first antibody (Genetex, AK, USA) and the second antibody (Santa Cruz Biotechnology, Dallas, TX, USA) were added, and DAB was then added to provide visualization. After conventional double-dye dehydration and mounting, each specimen was scanned (Pannoramic MIDI II, 3DHISTECH, Budapest, Hungary).

### Real-time cellular analysis (RTCA)

Cells were prepared at a concentration of 5 × 10^4^ cells/mL, about 50 μL of medium was added to the holes of an E-Plate 96 (ACEA Biosciences, CA, USA), and the E-Plate 96 was then placed on RTCA Station for baseline measurements. Then about 100 μL of the cell suspension was mixed evenly and added into the holes, so there were 8,000 cells/100 μL. After standing for 30 min, the sample was placed on an RTCA Station in the incubator overnight for measurement of cell proliferation.

### Acquisition of gene expression microarray data sets

Gene expression microarray data sets were acquired from the GEO database, from the National Center for Biotechnology Information (NCBI). Ninety-four data sets on lung metastases of OS were retrieved. After careful review and pre-treatment, GSE85537 and GSE37552 were selected for analysis of DEGs.

### Identification of differentially expressed genes

GEO2R, an interactive online analysis tool in R software (http://www.ncbi.nlm.nih.gov/geo/geo2r/) that uses the database of the NCBI [13], was used for analysis of DEGs. This tool was used to compare mRNA expression of OS specimens that had lung metastasis with OS specimens that had no lung metastasis. The criteria for a DEG: were: p < 0.05 and |log_2_FC|≥ 1.0. [14]. An online tool was used to create Venn diagrams that display similarities and differences. The DEG data were then downloaded as text files for subsequent analysis.

### Enrichment analysis of differentially expressed genes

Gene Ontology (GO) analysis was used for large-scale functional enrichment analysis and the Database Annotation, Visualization, and Integrated Discovery tool (DAVID, https://david.ncifcrf.gov/) was used for identification of standard gene names and functional interpretation [15]. GO analysis was used to classify genes by 3 functions: biological process (BP), molecular function (MF), or cellular component (CC). The Kyoto Encyclopedia of Genes and Genomes (KEGG) was used for further analysis of biological pathways related to the DEGs. All genes with significant upregulation or downregulation from comparisons were identified. A p-value below 0.01 and a count of 10 or more was considered significant.

### PPI network analysis and hub gene identification

The STRING database (https://string-db.org/) was used to search for known protein–protein interactions (PPIs) and to predict other protein–protein interactions. Version 11.0 of this database has information on 2031 species, 9,643,763 proteins, and 1,380,838,440 interactions [16]. The PPI network was visualized using Cytoscape software (www.cytoscape.org/), and the node in the center each plot was regarded as the core protein (or key candidate gene), a protein that has important physiological and regulatory function because of its high connectivity [17]. The CytoHubba plug-in for Cytoscape was used to calculate a score each protein node. The top seven DEGs based on correlation were identified as Hub genes.

### Survival analysis of hub genes

The Kaplan–Meier Plotter (http://kmplot.com/analysis/), an online tool that has data on 10,461 cancer samples, was used for survival analysis. This tool is continuously updated and integrates prognostic information from many databases to evaluate the effect of 54,675 genes on survival. The pan-cancer module in this tool was used. Patients were divided into a high-expression group and a low-expression group according to the expression of hub genes. Then, the prognostic value of the different hub genes was evaluated using the Kaplan–Meier method. The Bonferroni method was used to correct the p-value for multiple comparisons.

### Key genes verified by GEPIA: tissue level analysis

Gene expression in normal tissues and sarcoma tissues was also analyzed using the Gene Expression Profiling Interactive Analysis (GEPIA) tool, an interactive platform for integrating analysis and visualization of tumors and normal genes (http://gepia.cancerpku.cn/). The main data were from the Cancer Genome Atlas (TCGA) and RNA expression data of 9736 tumors and 8587 normal specimens of the genotype-tissue expression (GTEx) project [18].

### Statistical analysis

SPSS version 20.0 was used for statistical analysis. Data were expressed as means ± standard deviations. To assess the significance of differences, a *t*-test was used for comparisons of two groups and a one-way ANOVA for comparisons of multiple groups. The SNK-q test was used for pair-wise comparisons. Significance was set as fold-change of 2 or more and a P-value below 0.05. Pearson correlation analysis used to evaluate the relationship between POSTN levels and clinical parameters in patients with OS. Kaplan–Meier curves were used to analyze the effect of different parameters on survival.

## Results

### POSTN expression in different OS cell lines and tissues

**Fig. 1:**
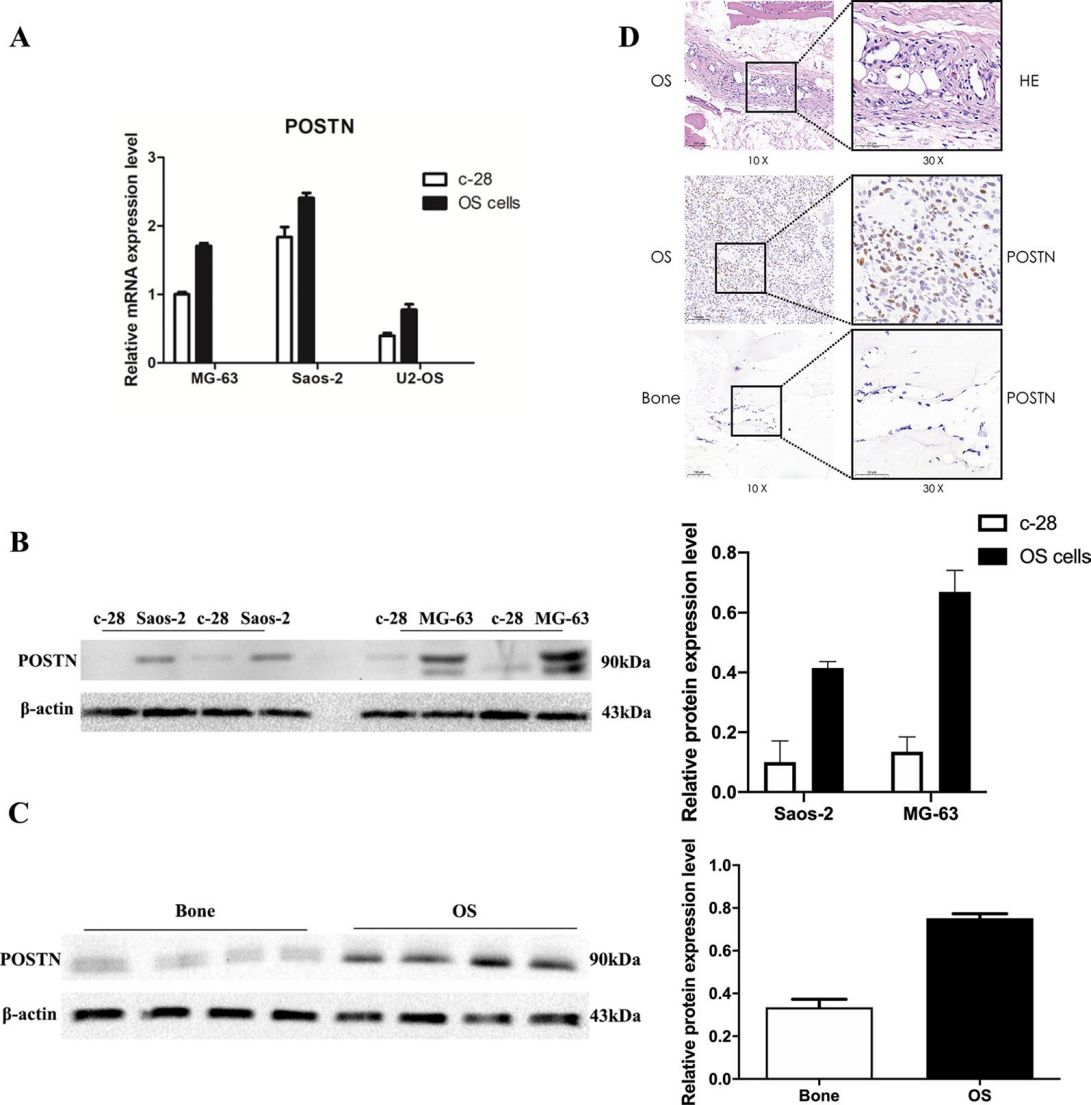
Expression of POSTN is greater in OS than normal bone cells. **A** qRT-PCR of POSTN in normal (C-28) and OS (MG-63, Saos-2, and I2-OS) bone cells (P < 0.0001). **B** Western blotting of POSTN in normal (C-28) and OS (MG-63 and Saos-2) bone cells (P = 0.007, P = 0.009). **C** Western blotting of POSTN in normal and OS bone tissues (P = 0.0014, n = 4). **D** Hematoxylin–eosin staining and immunohistochemical staining of POSTN in paraffin sections of normal and OS tumor tissues

We initially used qRT-PCR to compare the expression of *POSTN* in 3 different OS cell lines (MG-63, Saos-2, and U2-OS) with expression in a cell line of normal osteoblasts (C-28). The results indicated greater *POSTN* expression in all 3 OS cell lines (all P < 0.0001, Fig. 1A). Western blotting confirmed greater POSTN expression in 2 OS cell lines than in C-28 cells (both P < 0.01, Fig. 1B) and in 4 human OS tissues than in 4 adjacent normal bone tissues (P = 0.0014, Fig. 1C). Hematoxylin–eosin staining showed dense pink and polytypic osteoid tissues in the paraffin sections of OS tumors, and the immunohistochemical (IHC) results confirmed that POSTN expression was greater in OS tissues than in adjacent normal bone tissues (Fig. 1D).

### Effect of POSTN-shRNA knockdown on OS cells

**Fig. 2:**
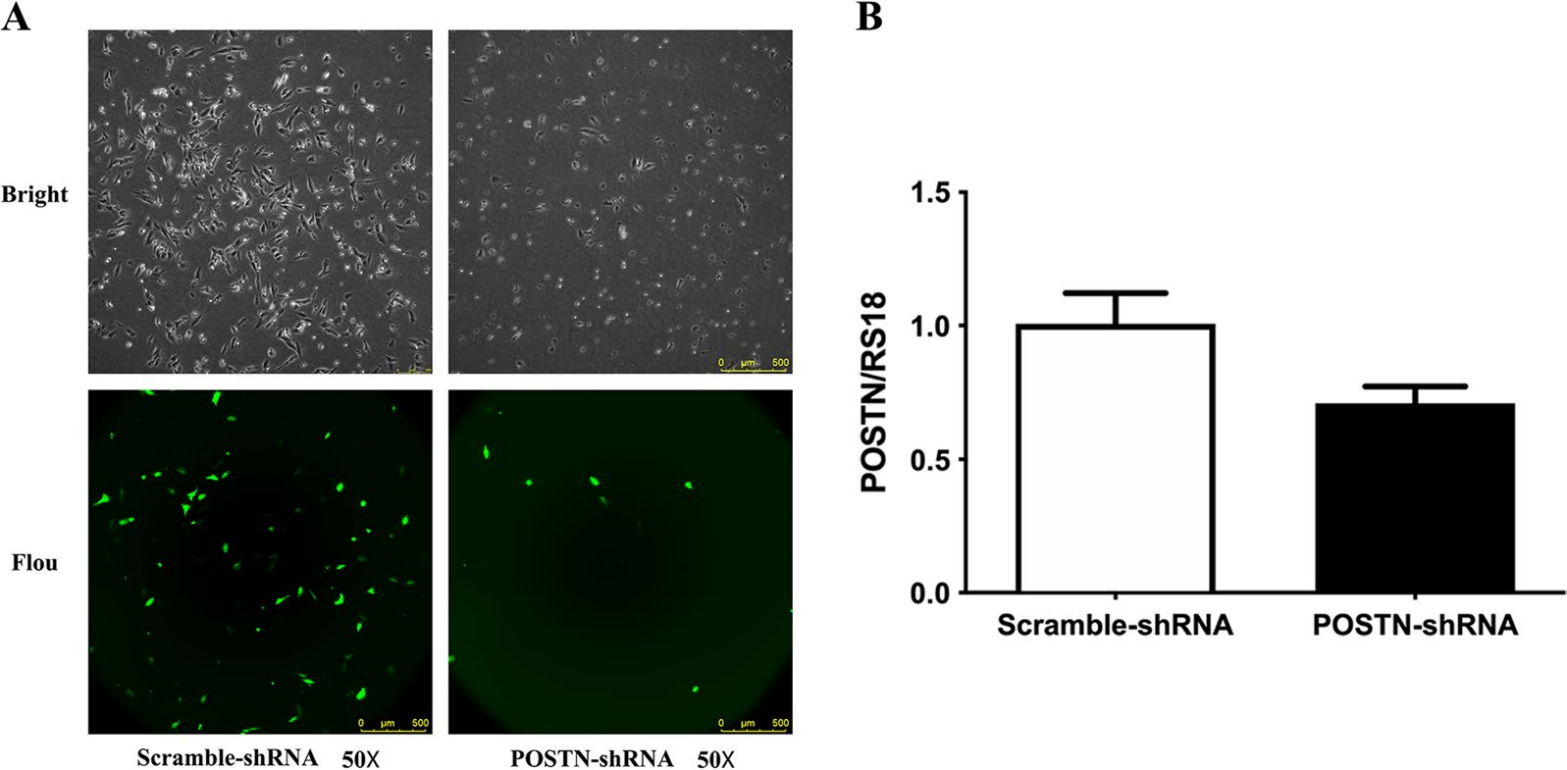
Transfection of OS cells with POSTN-shRNA downregulates POSTN expression. **A** Fluorescence microscopy of cells in the POSTN-shRNA and Scramble-shRNA groups (transfection efficiency: 60%). **B** qRT-PCR of POSTN in cells transfected with POSTN-shRNA or Scramble-shRNA (P = 0.017)

We used the Saos-2 cell line to examine the effect of *POSTN*-shRNA transfection on the expression of this gene. At 48 h after transfection, fluorescence microscopy indicated the transfection efficiency was 60% (Fig. 2A). Additional examination of some cells in each group using qRT-PCR indicated there was reduced expression of *POSTN* mRNA following *POSTN*-shRNA transfection (P = 0.017), thus confirming effective knockdown of this gene (Fig. 2B).

### Effect of POSTN knockdown on proliferation of OS cells

**Fig. 3:**
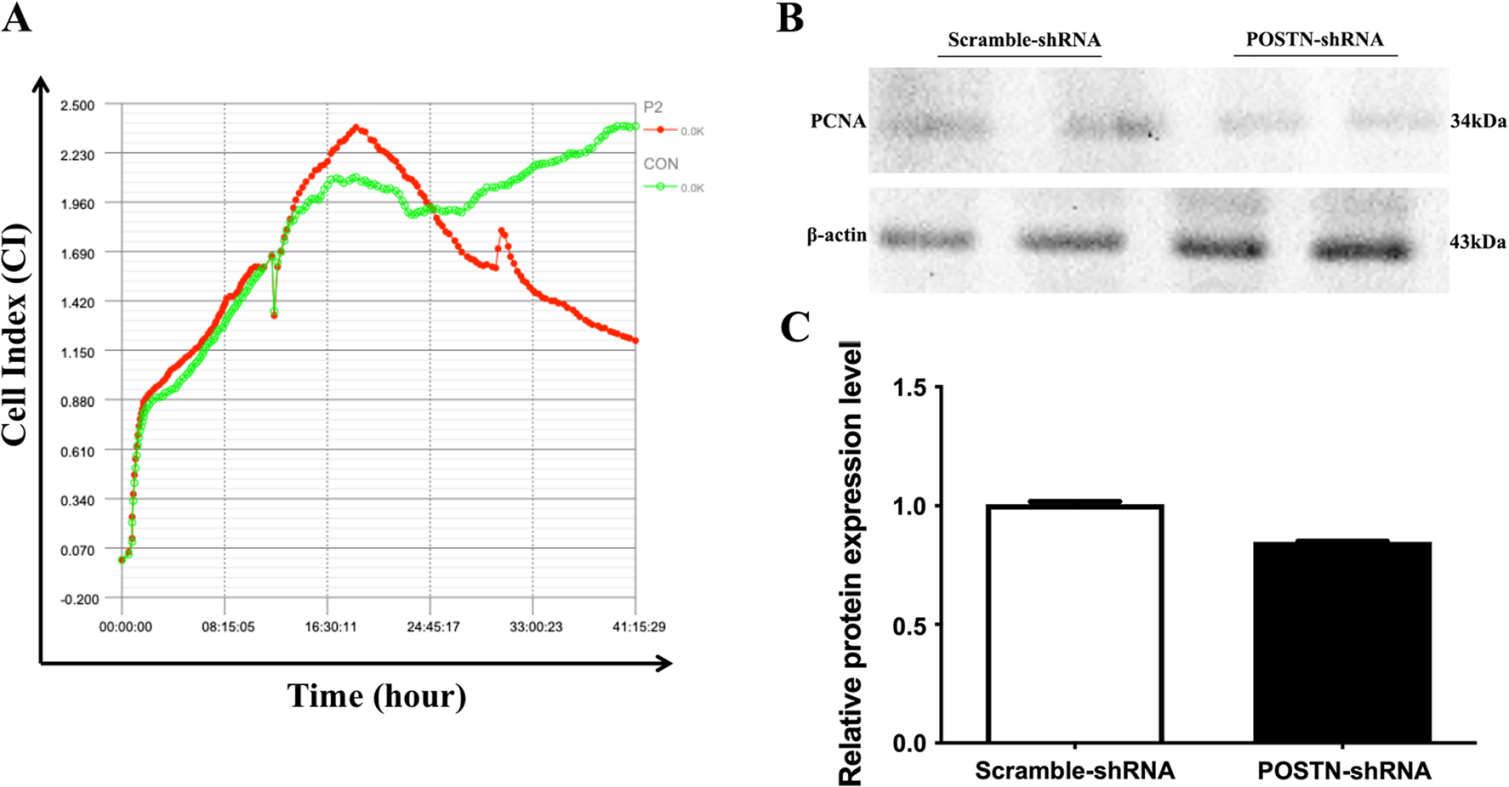
Transfection of OS cells with POSTN-shRNA inhibits cell proliferation. **A** RealTime Cellular Analysis of the proliferation of cells transfected with POSTN-shRNA or Scramble-shRNA. P2: POSTN-shRNA; CON: Scramble-shRNA. **B**, **C** Western blotting of PCNA expression in cells transfected with POSTN-shRNA or Scramble-shRNA (P < 0.0001)

We used label-free real-time cellular analysis (RTCA) to determine the effect of *POSTN*-shRNA on the proliferation of OS cells. At 24 h after transfection, cells in the *POSTN*-shRNA group had significantly reduced proliferation relative to cells in the *Scramble*-shRNA group (P = 0.0306, Fig. 3A). Examination of some cells in each group using western blotting indicated lower expression of proliferating cell nuclear antigen (PCNA, a marker of cell proliferation) in the *POSTN*-shRNA group than in the *Scramble*-shRNA group (P < 0.0001, Fig. 3B, C).

### Effect of POSTN knockdown on migration and invasion of OS cells

**Fig. 4:**
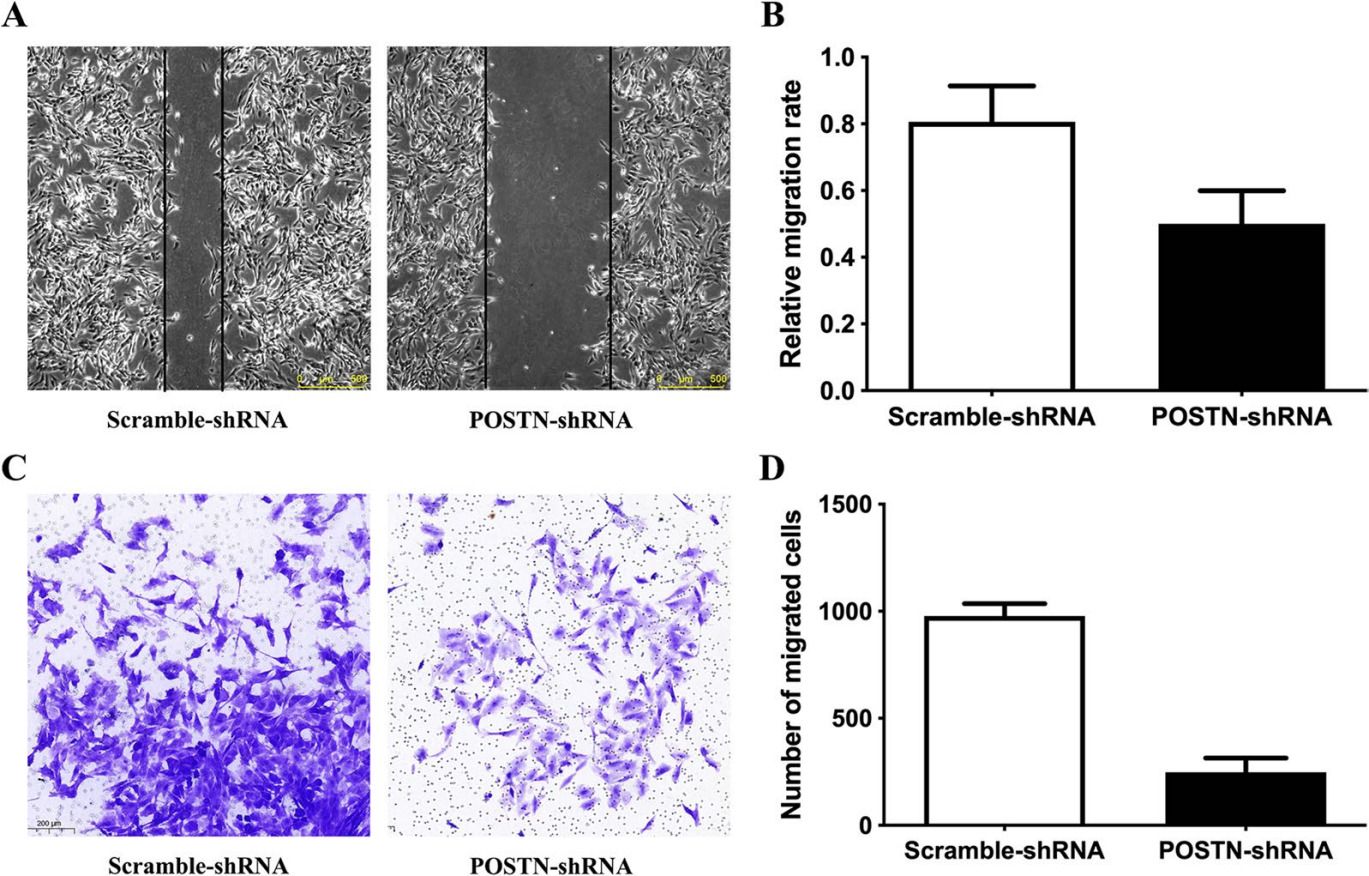
Transfection of OS cells with POSTN-shRNA inhibits cell migration and invasion. **A**, **B** Wound healing assay of OS cells transfected POSTN-shRNA or Scramble-shRNA (P = 0.0222). **C**, **D** Transwell assay of OS cells transfected POSTN-shRNA or Scramble-shRNA (P < 0.0001)

**Fig. 5:**
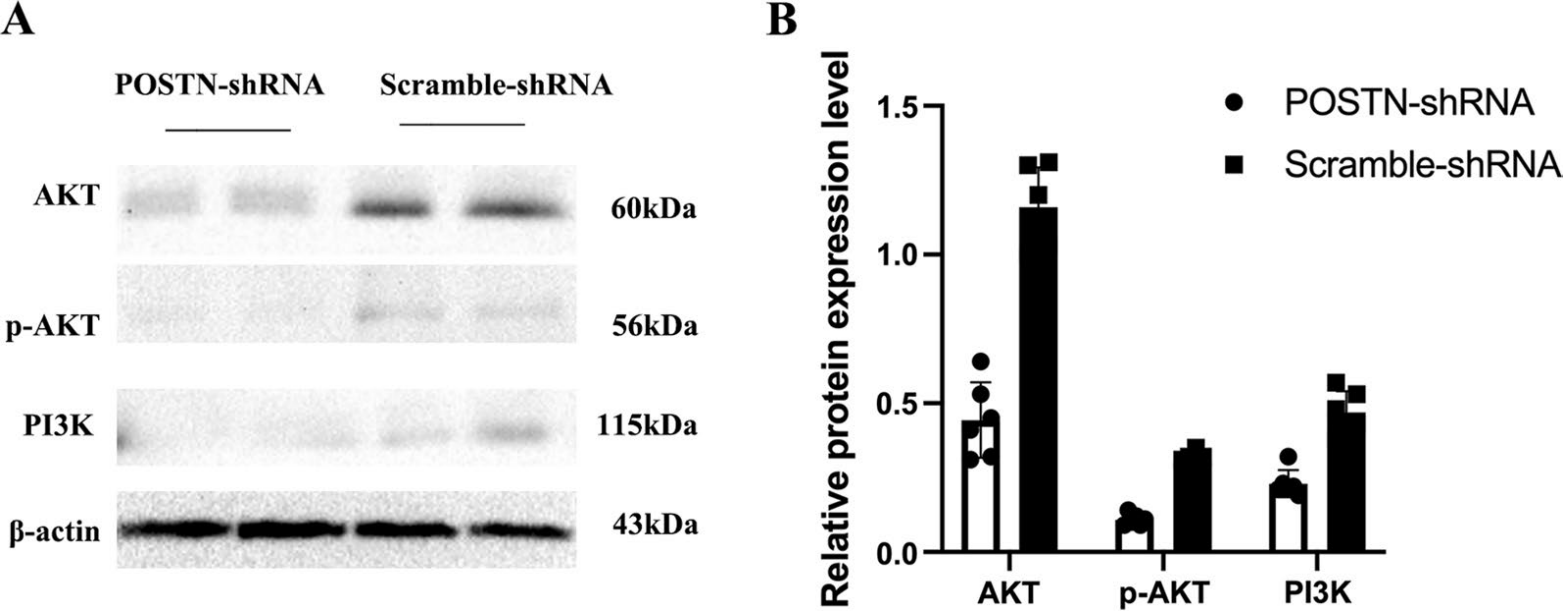
Transfection of OS cells with POSTN-shRNA downregulates the PI3K/AKT pathway. **A**, **B** Western blotting of AKT, p-AKT, and PI3K in cells transfected with POSTN-shRNA or Scramble-shRNA (all P < 0.0001)

We used the wound healing assay and the transwell assay to determine the effect of *POSTN*-shRNA transfection on the migration and invasion of Saos-2 cells. The results indicated significantly impaired cell migration (P = 0.0222) and invasion (P < 0.0001) in the *POSTN*-shRNA group relative to the *Scramble*-shRNA group (Fig. 4A, B). In addition, the western blotting of these cells showed that *POSTN*-shRNA reduced the levels of AKT (P = 0.0312), p-AKT (P = 0.0312), and PI3K (P = 0.0312), suggesting that PI3K/AKT signaling may function in OS progression (Fig. 5A, B).

### Expression of MMP-1, MMP-3, and MMP-13 in OS and normal bone tissues

**Fig. 6:**
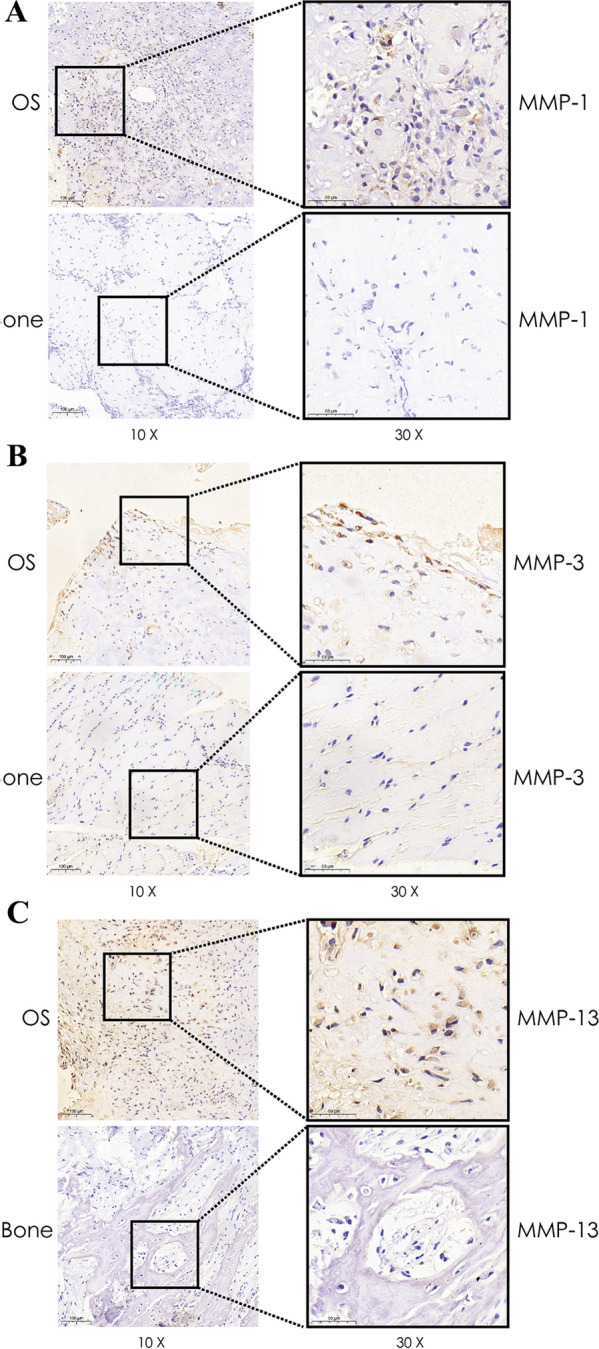
Immunohistochemical analysis shows that MMPs have higher expression in OS tissues. **A** MMP-1 expression in OS and normal bone tissue. **B** MMP-3 expression in OS and normal bone tissue. **C** MMP-13 expression in OS normal bone tissue

We performed immunohistochemical analysis to determine the expression of 3 different matrix metalloproteinases (MMPs, proteins that promote matrix disassembly) in OS and healthy bone tissues. The results indicated that OS bone tissues had increased expression MMP-1, MMP-3, and MMP-13 (Fig. 6A–C).

### Acquisition of gene expression data sets and identification of differentially expressed genes

**Fig. 7:**
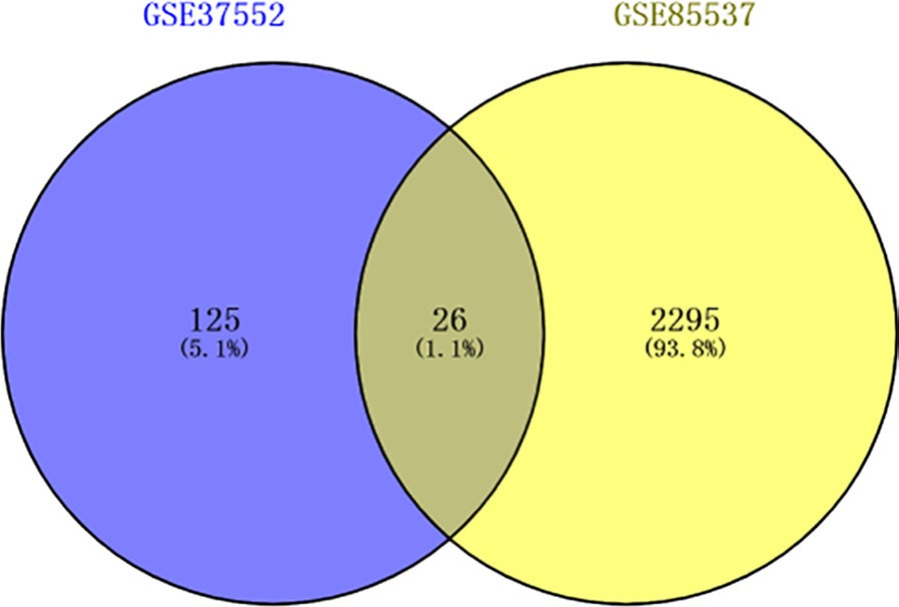
Comparison of gene expression in OS lung metastasis specimens with OS non-metastasis specimens (GSE85537 and GSE37552 datasets) indicates 26 significant DEGs

We reviewed 104 data sets of OS, and ultimately selected GSE85537 (containing 3 OS lung metastasis specimens and 3 OS non-metastasis specimens) and GSE37552 (containing 2 OS lung metastasis specimens and 2 OS non-metastasis specimens) for analysis of DEGs. The selected dataset was required to be ① a control sample containing the same number of lung and non-lung metastases of OSTEosarcoma ② a sample size of 4 or more ③ genetic information data was publicly available and available for analysis. Our comparison indicated that OS lung metastasis specimens and OS non-metastasis specimens had 26 significant DEGs (Fig. 7).

### GO and KEGG pathway analysis of differentially expressed genes

**Fig. 8:**
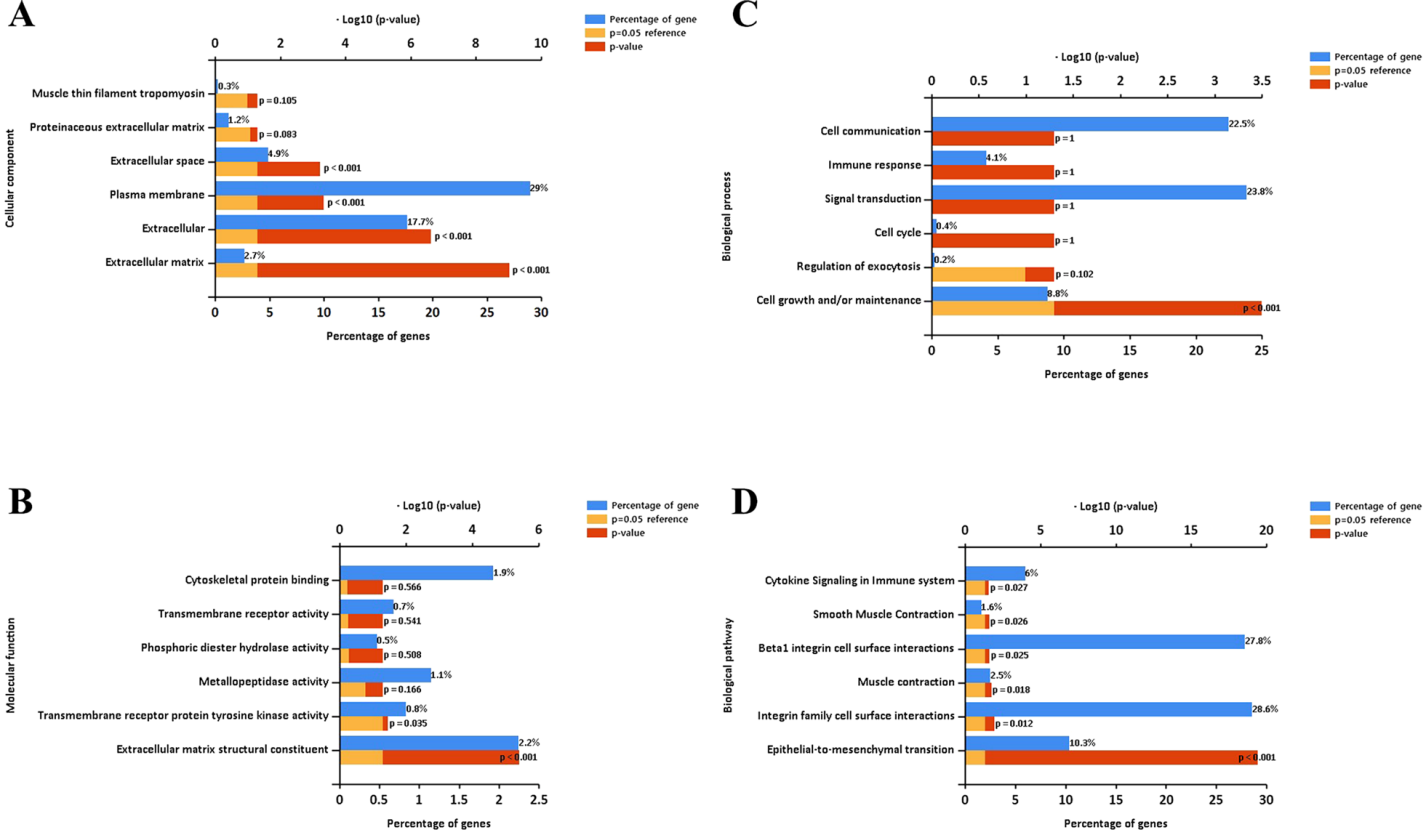
Functional gene enrichment analysis indicates OS tissues have DEGs in multiple categories. **A** Cellular components. **B** Molecular functions. **C** Biological processes. **D** Biological pathways

We performed GO and KEGG pathway analysis and used the Funrich tool to further analyze these 26 DEGs. The GO analysis classifies genes as functioning in biological processes, cellular components, or molecular functions. Our results indicated significant differences in the metastatic and non-metastatic OS specimens. In particular, at the level of biological process, upregulated genes in OS were related to extracellular mesenchyme, extracellular matrix, protein extracellular matrix, extracellular domain, membrane components, and myofibrillar myosin (Fig. 8A). At the level of molecular function, upregulated genes in OS were related to cytoskeleton protein binding, transmembrane receptor binding, phospholipid hydrolase activity, metallopeptidase activity, transmembrane receptor protein tyrosine kinase activity, and extracellular matrix structural components (Fig. 8B). At the level of biological process, upregulated genes in OS were related to cell junction, immune response, signal transduction, cell cycle, extracellular secretion regulation, and cell growth and maintenance (Fig. 8C).

### Protein interaction network and hub genes

**Fig. 9:**
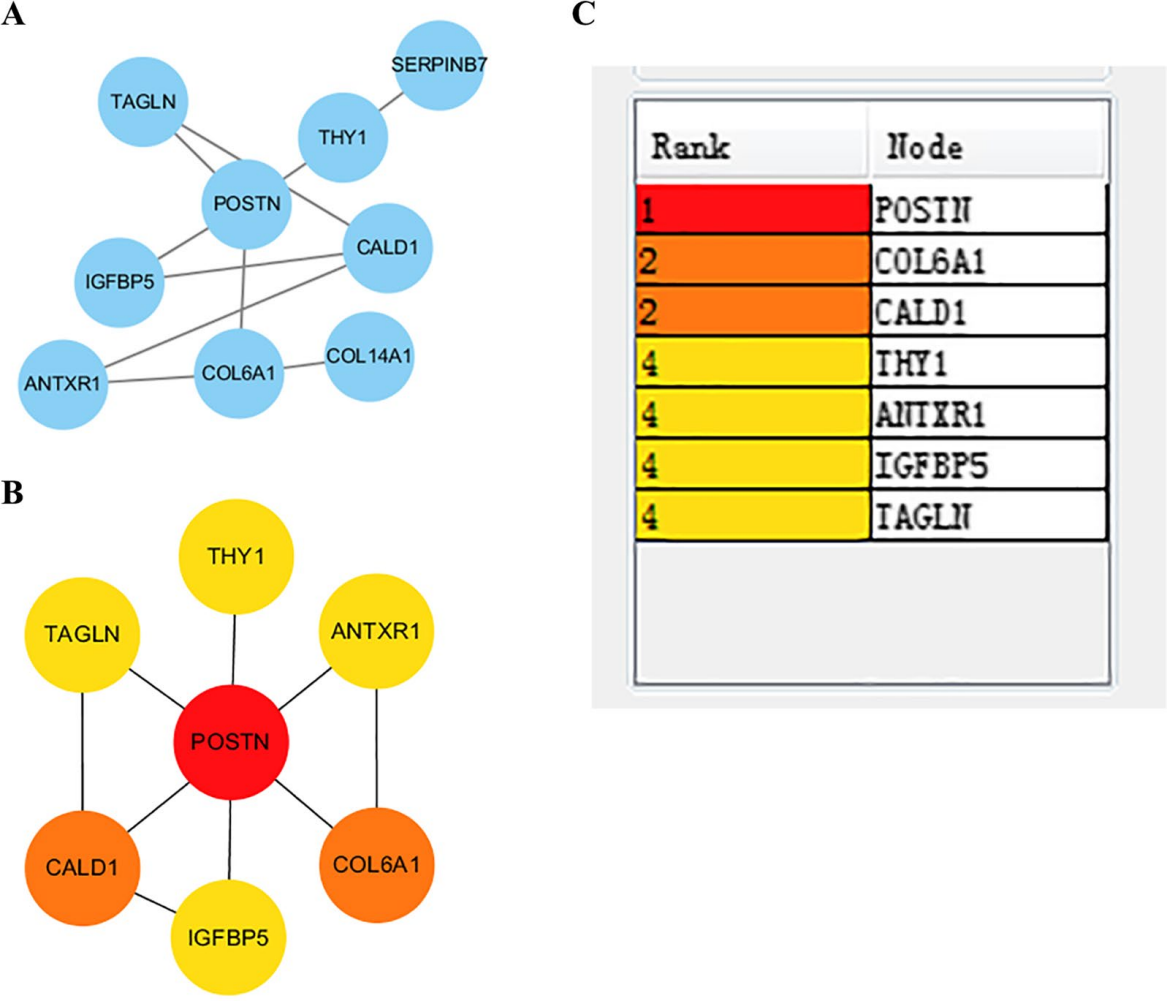
STRING analysis of direct and indirect protein–protein interactions in OS indicates that POSTN has the most interactions. **A** Co-expressed gene PPI network. **B** DEGREE results of the top 7 genes according to the degree of connection. **C** Connection ranks of the 7 major genes

We used the STRING tool to predict protein–protein interactions (PPIs) among the DEGs identified above (Fig. 9A), and then identified the top 7 genes using the cytoHubba template according to the degree of binding from the DEGREE algorithm (Fig. 9B, C). The first ranked gene was *POSTN*, and the other 6 major genes were collagen type VI alpha 1 chain (*COL6A1*), caldesmon (*CALD1*), thy-1 cell surface antigen (*THY1*), ANTXR cell adhesion molecule 1 (*ANTXR1*), insulin-like growth factor binding protein 5 (*IGFBP5*), and transgelin (*TAGLN*).

### Effect of POSTN expression on survival in sarcoma patient

**Fig. 10:**
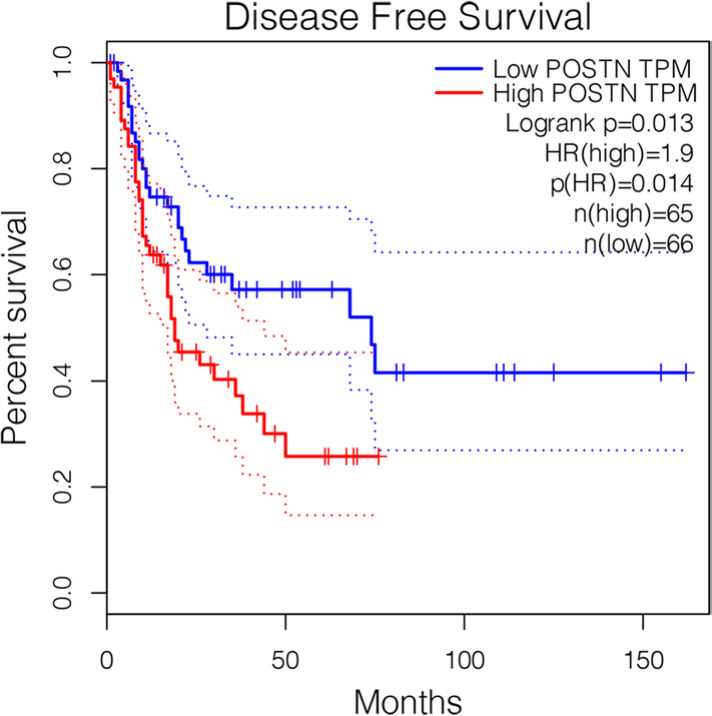
Kaplan–Meier analysis shows that OS patients with high *POSTN* expression have worse overall survival than those with low expression (P = 0.013)

Because the DEGREE algorithm ranked *POSTN* as the major gene, we used the Kaplan–Meier method to assess the relationship of *POSTN* expression on the prognosis of 131 patients with sarcoma. This analysis compared patients with high expression (n = 65) and low expression (n = 66) of *POSTN*. The results indicated that high expression of *POSTN* led to worse overall survival (P = 0.013, Fig. 10). Tissue level validation of key genes was performed using GEPIA to analyze the difference in expression between sarcoma and normal tissues. Explanation: At present, there is very little information about osteosarcoma in various tumor database platforms, and osteosarcoma is a major type of sarcoma, which also originates from mesenchymal tissue. Therefore, we believe that the above survival analysis results have certain reference significance.

## Discussion

OS is the most common primary malignant bone tumor and it mostly occurs children and teenagers [19]. Although this cancer is sensitive to some chemotherapeutic drugs, OS cells can develop drug resistance and this cancer has a tendency to form distant metastases [20]. OS tumors originate from interstitial cells, and they often metastasize during the early stage, mainly to the lungs. Since the 1990s, the introduction of neoadjuvant chemotherapy and targeted therapeutic drugs has greatly improved the survival rate of patients with early-stage OS. However, lung metastasis still occurs in about 50% of these patients and is one of the main causes of death [21]. Nonetheless, neoadjuvant chemotherapy combined with surgery remains the main clinical treatment for OS.

Although neoadjuvant therapy and surgery greatly improve the survival rate of patients with OS, the survival rate of these patients has not improved significantly in the past 30 years [19]. The 5-year survival rate of patients with metastatic OS is only about 20% [22]. The procollagen C-proteinase enhancer protein (PCOLCE) plays an important role in OS lung metastasis, and TWIST1 can slow the progression of metastatic OS by upregulating the expression of this protein [23]. Similarly, miR-223-3p can inhibit the invasion, migration, growth, and proliferation of OS cells by directly targeting CDH6. Hence, CDH6 can be used as a biomarker for metastatic OS [24]. Identification of additional new biomarkers and effective therapeutic targets is still necessary to improve the clinical treatment of OS. Because patients with metastatic OS have a survival rate of only 10 to 20% and those with non-metastatic OS have a survival rate of 50 to 78%, early diagnosis and prediction of metastasis is critical. We therefore performed the present bioinformatics analysis of in situ OS and lung metastasis, used the GEO online database to screen for signaling pathways and core genes, and used additional bioinformatics tools to further analyze these data sets. Our major result is that high expression of *POSTN* was associated with an increased risk of metastasis and poor prognosis in patients with OS.

*POSTN*, also called human osteoblast specific factor 2, was discovered by Takcshita in a mouse line of osteoblast cells. POSTN has an N-terminal signal peptide, a C-terminal hydrophilic domain, a cysteine rich region, and four internal homologous domains. This protein promotes osteogenesis and the aggregation and differentiation of precursor cells [25]. POSTN mainly occurs in connective tissues that have abundant collagen, has high expression in the periosteum and periodontal ligament, and is essential for bone formation and recovery from bone injury [26]. Previous in-depth studies of POSTN showed that it functions in cell recruitment, adhesion, and other normal physiological processes, but also has high expression in many malignant tumors [27–29]. For example, POSTN has high expression in breast cancer, and expression has a positive correlation with the pathological TNM stage; POSTN expression is also associated with lymph node metastasis in breast cancer. miR-876 targets *POSTN* and inhibits the epithelial mesenchymal transition (EMT) and fibrosis of hepatocellular carcinoma, thereby inhibiting the progression and prognosis of hepatocellular carcinoma. POSTN may therefore be an effective therapeutic target or prognostic marker for hepatocellular carcinoma [24]. There is also evidence that POSTN may regulate the EMT via the ERK signaling pathway, whose activation promotes the migration, invasion, and metastasis of tumor cells. Therefore, POSTN has potential as a therapeutic biomarker for hepatoblastoma [30].

The matrix metalloproteinases (MMPs), which are a family of enzymes that degrade the extracellular matrix, act as a vital role in the promotion of invasion and metastasis of OS [31]. MMP-1 over-expression in OS cells contributed to the proliferation, invasion, metastasis and stem-like properties of OS cells with the higher expression of VEGF and BMP 2/4 proteins [32]. Meanwhile, a higher MMP-3 expression in OS patients was found during tumor progression from primary to metastatic OS [33]. In addition, MMP-13 is closely related to the metastasis of OS and can be used as indicators to judge the metastasis of OS [34]. We found reduced expression of MMP1, MMP3 and MMP13 in human OS tissues and verified that by bioinformatics analysis.

POSTN also has high expression in OS tissues, and silencing the *POSTN* gene inhibits the angiogenesis of OS. Silencing of POSTN also inhibits the PI3K/AKT pathway, suggesting a role of this pathway in the pathogenesis of OS [11]. However, the relationship of POSTN with OS lung metastasis remains unclear. This led us to use the GEO database to analyze in situ OS and lung metastasis by screening data sets GSE85537 (3 OS lung metastasis samples and 3 OS non-metastasis samples) and GSE37552 (2 OS lung metastasis samples and 2 OS non-metastasis samples). We also used STRING to construct a PPI protein network of intersecting genes and used the DEGREE algorithm to select the seven genes that had the strongest correlations with surrounding genes. Our results indicated that *POSTN* ranked first. Our cellular and tissue level experiments confirmed the importance of *POSTN* in the pathogenesis of OS. In particular, we found that *POSTN* was highly expressed in OS cells and tissues, and silencing of *POSTN* by transfection of cells with *POSTN*-shRNA inhibited the proliferation of OS cells and also inhibited cell invasion and metastasis. Our results suggest that *POSTN* may play a role in OS pathogenesis by upregulating the PI3K/AKT signaling pathway.

In this study, we combined the use of GEO database analysis with in vitro experiments to verify high expression of POSTN in OS. Our results also indicated that silencing of *POSTN* inhibited the proliferation, invasion, and metastasis of OS. These findings have potential significance for the prognosis and treatment of OS. In particular, we suggest that *POSTN* be considered as a candidate gene for prediction of prognosis and lung metastasis in OS and as a potential therapeutic target for treatment of patients with OS.

## Data Availability

All data generated or analyzed during this study are included in this published article.

## Abbreviations

POSTN: Periostin gene
OS: Osteosarcoma
GEO: Gene Expression Omnibus
PPIs: Protein–protein interactions
RTCA: Real-time Cellular Analysis
NCBI: National Center for Biotechnology Information
GO: Gene Ontology
KEGG: Kyoto Encyclopedia of Genes and Genomes
GEPIA: Gene Expression Profiling Interactive Analysis
GTEx: Genotype-tissue expression
PCOLCE: Procollagen C-proteinase enhancer protein
EMT: Epithelial mesenchymal transition
